# Case of Occlusion of the Vagina, Nearly Complete, with Pregnancy and Labour

**Published:** 1842-01-29

**Authors:** N. T. Green

**Affiliations:** Danville, (Va.)


### *Case of Occlusion of the Vagina, nearly complete, with Pregnancy and Labour.* By N. T. Green, M. D.

To the Editors of the Medical Examiner.

Gentlemen,—Dr. Marsh's case of Atresia Vaginæ, published in the first number of your new series, has induced me to furnish you with one, which occurred in my practice during the past summer, very similar to it, except that the woman had previously borne children and was, at the time the case presented itself, pregnant and in labour.

Case.—On the 10th of August last, (1841,) I was requested to visit Martha, a negro woman about forty years of age, belonging to Mr. Price, whom I found sitting up, and complaining of nothing except a discharge of water from the vagina, which she said had come on suddenly a few hours before. She was pregnant, and believed herself to be in the seventh or eighth month. The discharge of water was not profuse, and there was no uterine pain. Her bowels were confined. She was directed to go to bed and open her bowels with a dose of castor oil. I called in the evening and found her quite easy. The oil had operated moderately. There was still a dribling of water from the vagina, not tinged with blood, and there was no pain. A dose of black drop was directed at night, and perfect quietude enjoined. This was done with the hope that she might still go many days longer, as I had known a case to go on a fortnight after the rupture of the membranes.

When I called next morning, contrary to my orders, she was again up, but felt no pain, and expressed herself as comfortable in all respects, except the discharge, which kept her wet. In the afternoon, however, I was sent for, and was told, by the old woman who was nursing her, that she was taken with pains shortly after my visit in the morning, which had been gradually increasing, and that, for some hours, there had been a slight discharge of blood from the vagina at each pain. On making an examination per vaginam, my finger came in contact with a globular body filling the upper part of the pelvis, having a slight depression in its centre—apparently the uterus with its mouth not sufficiently dilated to admit the end of my finger. By pressing firmly against the sides of this body, (which I frankly admit I then supposed to be the uterus,) I could feel the child higher up, and the sensation communicated to the finger was distinctly that of a spongy mass intervening between it and the head of the child. It instantly struck me that this was a case of attachment of the placenta over the os uteri, and hence the hæmorrhage; but a little reflection convinced me that such was not the fact;—for, in that case, there could have been no discharge of water until there was some detachment of the placenta; and, whenever this occurred, the waters must have become tinged with blood, which was not at all the case during the first day of their discharge. The pains, too, were apparently strong and forcing, and had been for some time, yet they had produced no effect in dilating what I then conceived to be the os uteri.

Unable to account satisfactorily for the appearances which presented themselves, and not seeing my course clearly, I determined to investigate the state of the parts thoroughly. With this view I placed the point of my finger in the depression supposed to be the os uteri, and, by pressing firmly, and rotating it on its point, I gradually, but not without some difficulty and force, succeeded in passing it through to about the first joint, when its tip came in contact with the child's head, and it was now manifest that the finger had passed through a membranous septum partitioning off the upper end of the vagina, with a very small opening in the centre, the edges of which still constricted the finger like a cord tied tightly round it. I could now readily ascertain the situation of the head—which presented properly—and of the os uteri—which was well dilated and soft. The vagina, too, above the septum, was capacious, and filled with coagulated blood, produced by its not escaping through the very small opening in the septum as fast as it was discharged from the uterus. This it was that produced the feel of a spongy mass intervening between the finger and head of the child at the first examination. With the finger which had been passed through it and the fore-finger of the other hand introduced into the vagina, I examined this septum minutely, and ascertained that it was a smooth, soft membrane, in thickness not more than two or three lines, equal throughout, in shape circular, and attached to the vagina transversely by its whole circumference. It was placed across the vagina rather more than an inch below the os uteri. After a careful examination I could detect no cicatrix or induration in the vagina, but every portion felt soft and dilatable.

The pains had now become brisk and forcible, and I determined to leave the septum untouched, in order that I might observe the manner in which the unaided efforts of nature would overcome the obstruction; knowing that I could at any moment divide it, should it present any serious impediment to the passage of the head. The head advanced rapidly, bringing the septum down before it almost to the os externum. When it began to press on the perineum, the membrane was drawn tightly over it, and the opening in its centre had dilated to the size of a dollar. In turning under the arch of the pubis, the opening dilated rapidly; but did not tear, so far as I could observe.

Another pain expelled the child, and the placenta followed in a few minutes. The child, which was small and apparently about the seventh month, was born asphyxiated; but, by great exertion, was revived, and afterwards grew and thrived well. The mother recovered rapidly, without any accident. Since her recovery I have had no opportunity of making an examination per vaginam, but shall do so if one offer.

This woman had previously borne several children, but none for the last ten years. In her two last labours before this, both of which were at full term, I attended her. They were both lingering and laborious, in consequence of disproportion between the child's head and the pelvis, which, in this woman, (who is herself of small stature,) is under the ordinary size. There was then no such formation as I have described existing in the vagina ; but it is probable that there might have been some inflammation and sloughing, consequent upon the last of these labours, (of which, however, I was not apprised,) which, in healing, may have given origin to this singular production. I am the more induced to adopt this solution of the difficulty in accounting for it, from the long immunity from pregnancy which this woman enjoyed ; for, unless we admit that conception may take place from absorption of semen in the vagina, this septum must have, almost completely, closed the door to that process.

*Danville, (Va.) Jan.*, 1842.

